# Chemical and physical compatibility of continuous intravenous drug infusion combinations used in paediatric intensive care

**DOI:** 10.1186/cc12321

**Published:** 2013-03-19

**Authors:** C Cole, A Fox, M Van Der Merwe, L Dickson, K Ball, A Bevan, A Hocking, J Pappachan

**Affiliations:** 1 University Hospitals Southampton, UK; 2 University of Portsmouth, UK

## Introduction

The aim of this research was to provide clinically relevant evidence for Y-site compatibility of drug infusion combinations used in the PICU. Pharmacists and clinicians regularly have to interpret limited published data, particularly when more than two drugs are Y-sited. The risk of potential incompatibility must be balanced against that of additional line insertion.

## Methods

A full 28-factorial design (total 256 combinations) was used to investigate chemical and physical compatibility of five drugs (clonidine, morphine, ketamine, midazolam and furosemide). The drugs were studied at their highest commonly infused concentrations and exposed to three variations in environmental conditions (diluent: sodium chloride 0.9% or glucose 10%; temperature 25 or 37°C; and normal room lighting or blue light phototherapy). Chemical stability was assessed using HPLC; >10% reduction in concentration indicated incompatibility. Physical incompatibility was confirmed by precipitation, pH or colour change.

## Results

Environmental conditions had no effect on the drug mixtures. The precipitation observed in incompatible combinations was due to either a change in pH, or with ketamine the presence of benzethonium chloride. Of 31 possible drug combinations, 12 were incompatible. A further three combinations were incompatible at extreme pH, or were of concern and so should be avoided. The incompatible formulations all contained furosemide. All combinations of the sedative agents studied were chemically and physically compatible.

## Conclusion

This work provides evidence for Y-site compatibility of morphine, midazolam, clonidine and ketamine in any combination, which will potentially reduce the need for extra intravenous lines. Furosemide is incompatible with any of these sedative drugs and must be infused via a separate line. These results will aid clinical decision-making and help satisfy the requirements of recent UK Department of Health legislation relating to the mixing of medicines.
